# Contribution of Testing Strategies and Contact Tracing towards COVID-19 Outbreaks Control: A Mathematical Modeling Study

**DOI:** 10.3390/tropicalmed7110376

**Published:** 2022-11-14

**Authors:** Shu-Chen Kuo, Byron Fan, Hongye Zhu, Meng-Hsuan Wu, Fang-Jing Lee, Yu-Chieh Cheng, Hsiao-Yu Wu, Ya-Ting Hsu, Chao A. Hsiung, Shiow-Ing Wu, Wei J. Chen, Hung-Yi Chiou, Huey-Kang Sytwu, Hsiao-Hui Tsou

**Affiliations:** 1 National Institute of Infectious Diseases and Vaccinology, National Health Research Institutes, Zhunan 35053, Taiwan; 2 Department of Applied Mathematics, Brown University, Providence, RI 02912, USA; 3 Department of Public Health, College of Public Health, National Taiwan University, Taipei 10617, Taiwan; 4 Institute of Population Health Sciences, National Health Research Institutes, Zhunan 35053, Taiwan; 5 Center for Neuropsychiatric Research, National Health Research Institutes, Zhunan 35053, Taiwan; 6 Institute of Epidemiology and Preventive Medicine, College of Public Health, National Taiwan University, Taipei 10617, Taiwan; 7 School of Public Health, College of Public Health, Taipei Medical University, Taipei 11031, Taiwan; 8 Master’s Program in Applied Epidemiology, College of Public Health, Taipei Medical University, Taipei 11031, Taiwan; 9 Graduate Institute of Biostatistics, College of Public Health, China Medical University, Taichung 40402, Taiwan

**Keywords:** COVID-19, SARS-CoV-2, universal testing, contact tracing, symptomatic screening

## Abstract

This modeling study considers different screening strategies, contact tracing, and the severity of novel epidemic outbreaks for various population sizes, providing insight into multinational containment effectiveness of emerging infectious diseases, prior to vaccines development. During the period of the ancestral SARS-Cov-2 virus, contact tracing alone is insufficient to achieve outbreak control. Although universal testing is proposed in multiple nations, its effectiveness accompanied by other measures is rarely examined. Our research investigates the necessity of universal testing when contact tracing and symptomatic screening measures are implemented. We used a stochastic transmission model to simulate COVID-19 transmission, evaluating containment strategies via contact tracing, one-time high risk symptomatic testing, and universal testing. Despite universal testing having the potential to identify subclinical cases, which is crucial for non-pharmaceutical interventions, our model suggests that universal testing only reduces the total number of cases by 0.0009% for countries with low COVID-19 prevalence and 0.025% for countries with high COVID-19 prevalence when rigorous contact tracing and symptomatic screening are also implemented. These findings highlight the effectiveness of testing strategies and contact tracing in reducing COVID-19 cases by identifying subclinical cases.

## 1. Introduction

In December 2019, the novel coronavirus disease (COVID-19) spread to over 100 countries within two months [1]. The etiologic agent was designated as severe acute respiratory syndrome coronavirus-2 (SARS-CoV-2) in January 2020. Cheong et al. [2] analyzed the advantages and disadvantages of implementing a lockdown versus maintaining an open community. Lai et al. [3] indicated that since COVID-19 is spread through close contact, many countries have adopted lockdown measures to contain its propagation at a tremendous socioeconomic cost. Cheong and Jones [4] suggested the outbreak has led to the collapse of global health systems, potentially triggering a public health crisis caused by opportunistic pathogens. Faced with this unprecedented challenge, public health authorities proposed universal testing or contact tracing with symptomatic screening as two possible solutions to mitigate the pandemic. Regarding the implementation of rigorous contact tracing along with symptomatic screening, numerous essential methods have been suggested. Kretzschmar et al. [5] proposed that the contact tracing or other mobile app technology is central to physical distancing. Aleta et al. [6] found that the response system with the enhanced testing and contact tracing are crucial factors in relaxing social-distancing interventions in the absence of herd immunity against SARS-CoV-2. Jian et al. [7] and Wang et al. [8] proposed that technology-assisted strict contact tracing with symptomatic screening could offer a cost-effective approach to contain the COVID-19 outbreak based on Taiwan’s experience. Libin et al. [9] proposed PCR testing of pooled households and further testing of COVID-positive groups to limit transmission.

Universal testing, another strategy to control the pandemic, could help identify asymptomatic infections. A modeling study demonstrated the role of asymptomatic infection in the transmission of COVID-19 possible at the population level. Pan et al. [10] pointed out that a large number of asymptomatic patients is the major factor contributing to the widespread COVID-19 pandemic and asymptomatic infection greatly affected the endemic equilibrium. To achieve the goal to decrease COVID-19 cases in an endemic equilibrium, the significance of controlling the asymptomatic infection should be stressed. Therefore, implementing effective containment policies to reduce asymptomatic infections is crucial. Furthermore, an article reviewing the epidemiological characteristics and prevention measures of asymptomatic infection with COVID-19 found that a lot of asymptomatic infections do not seek assistance from medical due to a lack of obvious clinical symptoms and awareness of prevention, which led to the rapid spread of COVID-19. Additionally, rigorous epidemiological investigations and laboratory testing are helpful in identifying asymptomatic infections, including follow-up surveys of the source of infection, close contact tracing, and testing of cluster epidemic [11]. Larremore et al. [12] suggested that frequent COVID-19 surveillance testing among asymptomatic individuals would lead to pandemic control and reduce reproductive number.

Universal or mass testing for COVID-19 is still being debated. Proponents of universal testing claim that universal testing is necessary to avoid the next wave and that mass testing will increase public confidence in the safety of reopening the economy. However, opponents of universal testing claim that mass testing is a waste of resources as it is time-consuming, labor-intensive, and unable to protect public people in a timely manner. Additionally, the high cost of universal testing may not be affordable for everyone [13]. For the applicability of universal screening, we are interested in investigating which testing strategies are the most effective in pandemic containment in areas with different population sizes prior to vaccine development, as it provides insight into the multinational containment effectiveness of the past and future emerging infectious diseases. Therefore, the aim of this research is to investigate if universal testing is necessary for containment when contact tracing and symptomatic screening measures are in place. Consequently, we explored the effectiveness of conducting testing strategies and contact tracing for SARS-CoV-2 based on currently known epidemiologic parameters. We assessed the ability of universal testing at various capacities in comparison with current contact tracing procedures and symptomatic screening policy.

## 2. Materials and Methods

### 2.1. Study Design

We used a stochastic transmission model, originally developed by Hellewell et al. [14] to assess the feasibility of controlling COVID-19 transmission through contact tracing and case isolation. We assessed the effectiveness of universal testing under the model assumptions employed by Tsou et al. [15]. For each COVID-19 patient, we assumed that both the incubation period and the delay from symptom onset to isolation followed a Weibull distribution (Supplementary Materials). Secondary cases were registered if the infected person had not been isolated by the time of likely infectiveness. We implemented a branching process model wherein the number of secondary cases consequent to each primary case was generated from a negative binomial distribution with a mean equal to the reproduction number R0. For each secondary case, we calculated the estimated transmission date based on the date of exposure to the primary case person and a serial interval generated from the skew normal distribution. The simulation of the model in this article focuses on the original strain of the coronavirus with the basic reproduction number (R0) as 2.5 [14]. Since the beginning of the pandemic, various SARS-CoV-2 variants have appeared across the globe. Currently, Omicron and its subtypes have become the most prevalent variants in many regions. Therefore, we also simulated the scenario of the SARS-CoV-2 Omicron variant with the basic reproduction number (R0) as 10 [16]. The details are documented in the Supplementary Materials. The key input parameters of the model are displayed in Table 1.

### 2.2. Simulation Scenarios

For the untraced infectious cases, there are two possible scenarios:

1. Symptomatic Screening: We assumed that all symptomatic cases are immediately presented to clinics for RT-PCR testing.
  - We further simulated the scenarios that either 30%, 50%, 70%, or 100% of symptomatic cases are immediately presented to clinics for RT-PCR testing. We simply designated “30%, 50%, 70%, or 100% of symptomatic screening” to represent the corresponding scenarios.
  - We assumed sufficient testing capacity to accommodate all cases presented to the clinics.
2. Universal testing: We assumed that asymptomatic and subclinical cases have the potential to obtain universal testing based on the proportion of total testing capacity to the population size. Due to the severity of outbreaks in the United States (USA) and United Kingdom (UK), we assumed that both countries reach their maximum testing capacities each day. Current universal testing capacities are approximately 0.5% of the total population per day in the USA and 1% of the total population in the UK [26,27]. However, current universal testing capacities in some Asian settings such as Taiwan and the Republic of Korea (South Korea) are approximately 0.05% and 0.03% of the total populations per day, respectively [25,28]. We chose to analyze the 0.5% capacity in the USA as a representation of current universal testing capacity. Therefore, the current and projected universal testing capacity scenarios analyzed are as follows:
  - Current universal testing capacity: 0.05% to 1% [25,26,27,28].
  - Projected universal testing capacity: 5% to 10% [29].

**Figure 1:**
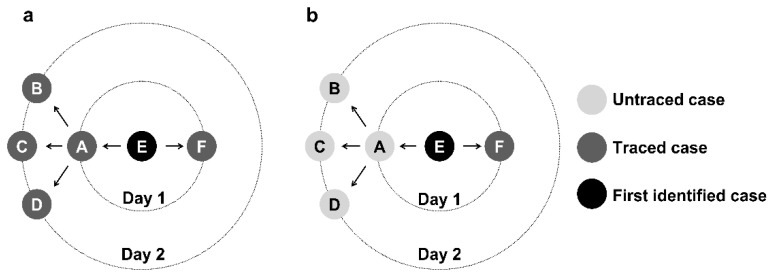
Contact tracing procedure for individual COVID-19 cases labeled A-F. A infects B, C, D, and E. Then E infects F. We assume E is the first identified case. Through contact tracing, cases A and F might be identified. On the next day, cases B, C, and D might be traced following the same tracing procedure through the new confirmed case A. (**a**) Successful tracking scenarios. We assume the tracking success rate is 80%; (**b**) Unsuccessful tracking scenarios. An intermediate untraced case will prevent contact tracing from identifying further secondary cases.

For the implementation of contact tracing, we assumed that a proportion of contacts infected with COVID-19 can be identified on the first day after confirming the primary case (contact tracing success rate in Table 1). The same contact tracing procedure was subsequently applied to new confirmed cases. We assumed that confirmed cases were isolated immediately and removed from further transmission. Figure 1 illustrates (a) Using the simulated transmission process in Figure S2 in the Supplement, the simulated contact tracing began with the confirmation of infectious case E (black) with an 80% success rate of tracking each case. Through contact tracing, cases A and F (dark gray) were successfully traced. On the next day, cases B, C, and D (dark gray) were traced following the same tracing procedure through the new confirmed case A; (b) An intermediate untraced case will prevent contact tracing from identifying subsequent secondary cases. Failure to trace case A (light gray) results in failure in contact tracing for cases B, C, and D (light gray).

### 2.3. Control Strategies

The reference scenario was defined when no intervention was applied (Reference). With no interventions (i.e., contact tracing success rate at 0%) as our reference scenario (Reference), the control strategies could be: (I) contact tracing at 40% or 80% success rates with no additional intervention, (II) strategy I and additional symptomatic screening, and (III) strategy II and either universal testing at current capacities of 0.05%, 0.5%, and 1%, or projected capacities of 5%, and 10% of the total population per day.

### 2.4. Outbreak Control

Let $n_{i}$ be the total number of cases on the $i^{th}$ day, and $n_{i-1}$ be the total number of cases on the ${\left(i-1\right)}^{th}$ day. The strict definition of outbreak control is that the daily increase of cases is less than one ($n_{i}-n_{i-1}<1$) for at least 7 days. A “light” outbreak is defined as a daily increase of cases that is less than ten ($n_{i}-n_{i-1}<10$) for at least 7 days. In reference to the study by Hellewell et al. [14], the uncontrolled outbreaks were defined as reaching 5000 cumulative cases and were assumed too large to be controlled in 40 days.

### 2.5. Definition of High/Low Prevalence Rate Countries

High COVID-19 risk is defined by a daily incidence rate at or above 10 cases per 100,000 people [30]. Therefore, for the simulation, a group with a population of 2,000,000 and an initial case number of 20 on day 0 is defined as having a low prevalence rate. In contrast, a group with the same population and 200 cases on day 0 is defined as having a high prevalence rate.

### 2.6. Simulations

**Figure 2:**
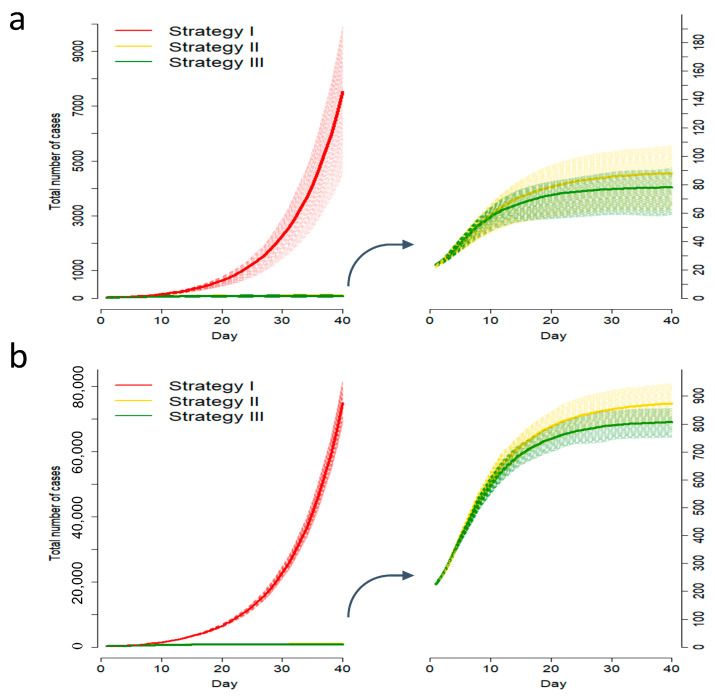
Simulated total number of cases under different control strategies. (**a**) The average number of cumulative cases in a low prevalence country; (**b**) The average number of cumulative cases in a high prevalence country. The shaded region of figures (**a**,**b**) represents the inter-quartile region for different strategies.

The simulation study was performed for various combinations of input parameters such as the number of initial cases, contact tracing success rate, current and projected universal testing capacities, proportion of pre-symptomatic transmission, delay from symptom onset to isolation, percentage of subclinical cases, incubation period, number of secondary infections generated by each new infection, and serial interval (Table 1). The total number of cases for three control strategies (I), (II), and (III) were simulated. The comparisons among the control strategies are presented in Figure 2. Figure 2a,b show that strategy (I) cannot control an outbreak. The total number of cases drops dramatically by conducting control strategy (II) through the additional one-time symptomatic screening of all symptomatic patients. In comparison of strategy (III) at a 0.5% testing capacity with strategy (II), Figure 2a shows that there is only a 0.0009% decrease in the total number of cases in 40 days, indicating a minimal difference. Figure 2b shows a 0.025% decrease in the total number of cases in 40 days (case number decreases from 860 to 779), indicating a distinguishable difference in a high prevalence country.

Case reduction is defined as the percentage of case decrease relative to the Reference on day 40. More specifically, let $N_{Ref}$ and $N_{j}$ be the total number of cases on the 40th day under the scenarios of the Reference and strategy *j*, respectively, the percentage of case reduction of strategy *j* from the Reference is defined as $(N_{Ref}-N_{j})/N_{ref}$. The percentage of case reduction compared to the Reference on the 40th day for three control strategies (I), (II), and (III) were simulated.

## 3. Results

Our article mainly focuses on the simulation results of the original virus strain and pro-vides a few simulation results of the SARS-CoV-2 Omicron variant scenario.

### 3.1. Low Prevalence Countries

#### 3.1.1. Countries with 40–80% Contact Tracing Success Rates

**Figure 3:**
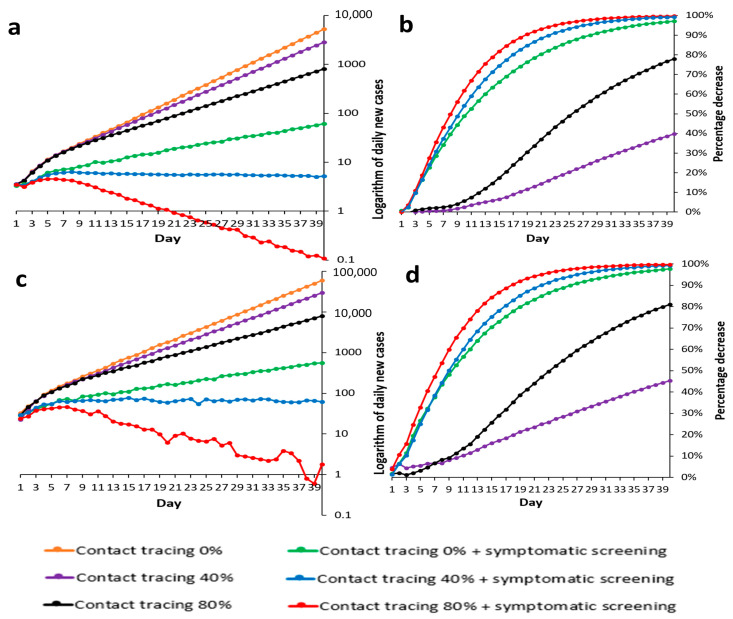
Logarithmic curves of daily new cases & percentage decrease of daily new cases in contexts of different contact tracing and symptomatic screening. (**a**) Daily new cases in a low prevalence country; (**b**) Percentage decreases of daily new cases in a low prevalence country compared to the Reference; (**c**) Daily new cases in a high prevalence country; (**d**) Percentage decreases of daily new cases in a high prevalence country compared to the Reference.

Figure 3a shows logarithmic curves of simulated daily new cases, and Figure 3b shows each strategy’s percentage case decrease. Contact tracing alone did not control outbreaks; the case reduction rates were 39.7% and 77.9% with 40% and 80% contact tracing success rates, respectively. The addition of symptomatic screening increased effectiveness, compared to 40% and 80% contract tracing alone, to 99.3% and 99.7% case reduction, respectively. Symptomatic screening combined with 80% contact tracing achieved outbreak control by day 27 (Table S1). When this strategy was applied with universal testing at a 0.5% capacity, the case reduction rate increased by only 0.0009% (from 99.7373% to 99.7382%). Increasing the universal testing capacity to 1%, 5%, and 10% shortened the delay to outbreak control by only 1, 3, and 5 days, respectively (Table S2). These results suggest that for countries with low prevalence, universal testing is unnecessary if 80% successful contact tracing and symptomatic screening policies are in place. From the analysis of the daily case increase under these two scenarios, 40% successful contact tracing with symptomatic screening failed to achieve outbreak control within 40 days, which could be achieved only with the addition of >5% universal testing (Table S3).

#### 3.1.2. Countries with No Contact Tracing

**Figure 4:**
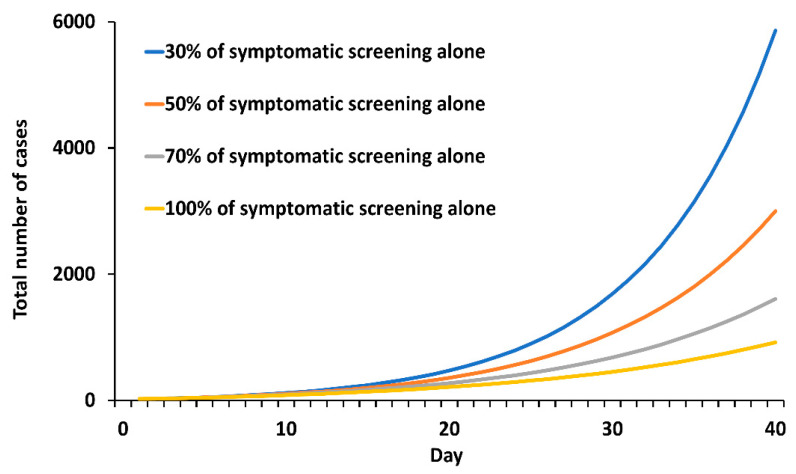
Simulated total number of cases under symptomatic screening alone.

Simulated results showed that 30%, 50%, 70%, and 100% of symptomatic screening alone led to an 82.2%, 90.9%, 95.1%, and 97.2% case reductions, respectively, compared to the Reference (Figure 4). Outbreak control for full symptomatic screening (that is, 100% of symptomatic screening) required ≥15% universal testing capacity (Table S4).

#### 3.1.3. The SARS-CoV-2 Omicron Variant Scenario

Simulated results show that a universal test at 0.5% coverage with no contact tracing led to more than 5000 infections after day 30 (Table S9). The result showed the epidemic cannot be controlled in the short term. The 0.5% universal test is a high screening capacity, requiring a lot of manpower, involving human privacy right concerns, and significant eco-nomic impacts. In the omicron variant period, it is no longer suitable to judge the number of cases to determine the breakout control. To maintain the new normal lifestyle, only the administration of vaccines can slightly reduce the number of confirmed cases and greatly reduce the number of severe cases and deaths, developing a coexistence lifestyle with the coronavirus.

### 3.2. High Prevalence Countries

#### 3.2.1. Countries with 40–80% Contact Tracing Success Rates

Contact tracing at 40% and 80% alone led to 45.3% and 80.9% case reduction rates, respectively (Figure 3c,d), which were insufficient to achieve outbreak control (Table S5). The addition of symptomatic screening to contact tracing of 40% and 80% improved case reduction rates to 99.3% and 99.8%, respectively. However, neither of the two strategies could achieve outbreak control (Table S5). With 80% contact tracing, symptomatic screening with the addition of 5% and 10% universal testing coverages shortened the duration needed to control the outbreak to 35 and 27 days, respectively (Table S6). With contact tracing at 40%, symptomatic screening could not achieve outbreak control with universal testing at 5% or 10% coverage (Table S7).

#### 3.2.2. Countries with No Contact Tracing

Full symptomatic screening (that is, 100% of symptomatic screening) led to a 97.6% case reduction (Figure 3c,d). The addition of universal testing at 0.5%, 5%, 10%, 15%, and 20% coverage of the total population per day enhanced case reduction rates to 98.2%, 99.3%, 99.6%, 99.7%, and 99.8%, respectively. However, these combined strategies were insufficient to gain control of transmission (Tables S5 and S8). Moreover, simulated results show that 30%, 50%, and 70% of symptomatic screening alone led to 83.3%, 92.0%, and 94.9% case reductions, respectively, compared to the Reference (Figure S3).

### 3.3. Time to Outbreak Control with 100% or 50% Symptomatic Screening

**Figure 5:**
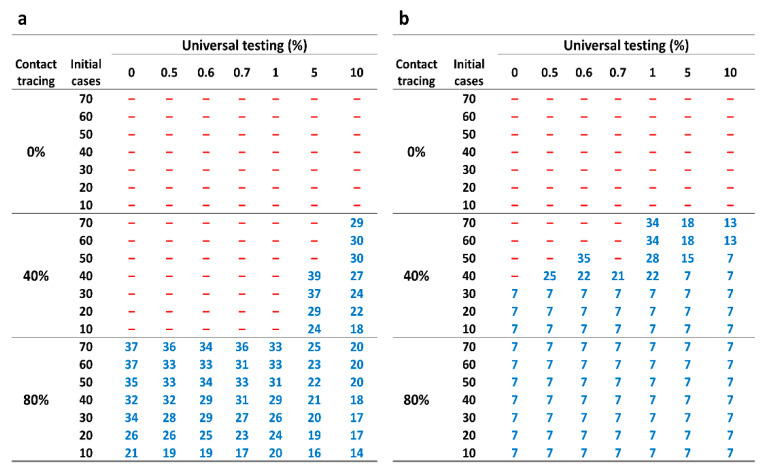
Time to outbreak control with 100% symptomatic screening. This figure shows the number of days to outbreak control for the simulated COVID-19 pandemic with 0%, 40%, and 80% contact tracing and 100% symptomatic screening. The earliest day to achieve outbreak control is based on (**a**) the strict definition; (**b**) the light definition. **–**: The epidemic is not controlled at any time.

Figure 5 shows outbreak control using the strict and light definitions in different settings. For example, the simulated COVID-19 transmission achieved outbreak control by day 29 and day 7 for strict and light definitions, respectively, for the strategy with 40 initial cases, 80% contact tracing, 100% symptomatic screening and 1% universal testing.

Furthermore, increased symptomatic screening can achieve earlier outbreak control for simulated COVID-19 transmission. Under 40 initial cases, 80% contact tracing and 5% universal testing in the strict definition, the intervals to achieve outbreak control are 23 and 21 days, respectively, for simulated COVID-19 transmission for 50% symptomatic screening (Figure S5) and 100% symptomatic screening. Under the same settings using the light definition, the corresponding intervals are both 7 days.

Outbreak control when using the strict definition for 50% symptomatic screening is difficult to achieve in the lower contact tracing and lower universal testing simulations (Figure S5). For example, if the initial case number is 10 with 40% contact tracing and 0.5% universal testing, the outbreak is uncontrolled. However, if universal testing is increased to 5%, the outbreak can be controlled using the strict definition. Furthermore, higher universal testing rates such as 10% in conjunction with 40% level of contact tracing using the strict definition show that outbreaks with 10–70 initial cases are easily controlled.

## 4. Discussion

Our study suggests that in populations with either low or high COVID-19 prevalence, testing strategies and contact tracing are effective at identifying subclinical cases, resulting in an overall case reduction. Moreover, our findings highlight the importance of implementing testing and maintaining non-pharmaceutical interventions such as social distancing and contact tracing. Our simulations demonstrate that the importance of testing rises as national capacities to trace symptomatic individuals decline. Increased universal testing and symptomatic screening facilitate earlier outbreak control during the ancestral SARS-Cov-2 virus propagation. Our simulation may inform outbreak control through the combination of contact tracing, universal testing, and symptomatic screening (Figure 5 and Figures S4–S6). It enables governments to evaluate COVID-19 transmission under different policies of contact tracing, universal testing, and symptomatic screening.

The findings of our simulation are concordant with the analysis of the Belgian COVID-19 epidemic conducted by Libin et al. [9], who found that outbreak control may be feasible through universal testing, even in the context of looser contact tracing abilities. Innate difficulties in identifying contacts in crowded urban settings and insufficient manpower of government will pose challenges for contact tracing [31]. The government is committed to ensuring that all people at risk are quarantined, which is time-consuming and labor-intensive [8]. Furthermore, in an executive summary of universal testing by Johnson-Leon et al., the benefits of frequent testing highlighted the importance of universal testing as a complement to other mitigation strategies, which supports our scenarios of combining universal testing with other non-pharmaceutical interventions at varying success rates to match different national capacities [32]. Currently, countries that implement testing utilize a prioritization strategy that evaluates health benefits [33], whereas our model used a uniform approach to testing for universal testing strategies.

The success of testing is due largely to the management of obtaining true-positive results. This process is comprised of testing, confirmation of positive results, and isolation to reduce the probability of COVID-19 transmission during the infectious stage. However, because the sensitivity of testing was 0.777, varying from 0.671 to 0.875 [23], there is an issue of false negatives. This would present an inability to isolate all SARS-CoV-2-infected individuals to prevent transmission to other members of the population. Collaboration with diagnostic device manufacturers may facilitate the creation of more accurate PCR tests or to switch towards newly developed rapid COVID-19 testing systems.

We have also designed our statistical model to be applicable to countries at different stages of pandemic preparedness. Our goal is to provide support for decision-making to public health officials by investigating potential case reductions utilizing combinations of universal testing and non-pharmaceutical interventions. This agent-based model can be adapted for countries with varying levels of contact tracing capacity and COVID-19 testing infrastructure. However, depending on the technology and public information utilized by governments, our approach may be less accurate for populations that are less willing to partake in COVID-19 mitigation measures.

Our findings suggest that in countries with low COVID-19 prevalence, rigorous contact tracing with symptomatic screening can be sufficient to control outbreaks, obviating the need for universal testing. In the context of a low COVID-19 prevalence with relatively limited contact tracing, symptomatic screening and universal testing were found to be required to achieve outbreak control. However, in countries with a high COVID-19 prevalence, achieving outbreak control required rigorous contact tracing with symptomatic screening and high-coverage universal testing (Table 2).

## 5. Limitations

Our simple model has notable limitations. We assumed that there would be no delay from the moment of symptom onset to symptomatic testing. Similarly, we assumed that all symptomatic screening tests would be available immediately upon request. By excluding factors of each country’s limited resources and manpower, these assumptions may lead to an underestimate of pandemic trajectories in the simulations. While this analysis offers insight for the necessary non-pharmaceutical interventions and testing in any country’s population, it does not speak to the challenges regarding the implementation in each population, which include public adherence to guidelines, resource acquisition, quarantine measures, and in-hospital treatments. In addition, we did not explicitly model close contact, i.e., among household members. Another limitation is that our simulation scenarios do not account for heterogeneity within the population. Tremendous variations can occur across individuals and geographic regions with respect to predisposing conditions, compliance with pandemic control measures, and, consequently, susceptibility to and transmissibility of the SARS-CoV-2 virus. Although population characteristics, non-pharmaceutical interventions, and testing scenarios do not specifically match a particular country’s demographics, our model has the advantage of quantitatively simulating outcomes associated with the implementation of such COVID-19 case reduction measures.

## 6. Future Work

We will explore the random time period to represent either the delay between symptom onset and symptomatic testing or the delay between symptomatic testing and the acquisition of the COVID-19 report request. For the heterogeneity within the population, we will adjust the parameters of disease transmission as it varies across each age-group or a specific population group in the country. Moreover, we will explore each transmission scenario when the initial cases are composed of various COVID-19 symptoms severity categories, such as asymptomatic, mild, moderate, severe, and critical. We will also adjust the reproduction number based on the COVID-19 symptoms severity composition of initial cases. In addition, we will consider the intervention of vaccination, such as the percentage coverage of population received vaccine, the vaccine efficiency of 2-dose and 3- dose series, and the level of reduction of vaccine efficiency across time. By considering these new parameters, we will develop a suitable model considering different scenarios of SARS-CoV-2.

## 7. Conclusions

The results of our decision analysis model suggest that universal testing should be reserved for countries with high COVID-19 prevalence rates or those with minimal contact tracing capacities. We discovered that for countries with low prevalence rates, with premises of strict enforcement of contact tracing at a success rate of 80% and rigid implementation of symptomatic screening, current universal testing capacities would become insignificant. However, for countries with high prevalence rates, with 80% contact tracing, symptomatic screening with the addition of 5% and 10% universal testing coverages shortened the duration needed to control the outbreak to 35 and 27 days, respectively. Thus, for countries with low prevalence rates and limited/no contact tracing capacities, or for those with high prevalence rates, increased contact tracing success rates and expanded universal testing capacities are necessary to contain the COVID-19 pandemic. Therefore, the value of testing strategies and contact tracing in these settings lies within its ability to identify subclinical cases. These findings emphasize the importance of focusing efforts on implementing non-pharmaceutical interventions in all countries.

## Figures and Tables

**Table 1:** Key model input parameters.

Sampled	Mean (SD), n or %.	Reference
Delay from symptom onset to isolation	9.76 days (7.66)	Liu et al. [17]; Tsou et al. [15]
Incubation period	5.8 days (2.6) *	Backer et al. [18]
	3.24 days (0.8) **	Helmsdal et al. [19]
Serial interval	5.8 days (2) *	Hellewell et al. [14]
	3.64 days (2.16) **	UKHSA [20]
**Fixed**		
Initial cases	20, 200	Assumed
Contact tracing success rate	0%, 40%, 80%	Assumed
Reproduction number (R0)	2.5 *	Hellewell et al. [14]
	10 **	Talha KhanBurki [16]
Percentage of subclinical cases	40% *	Oran & Topol [21]
	23% **	Garrett et al. [22]
Probability of pre-symptom transmission	55%	Casey et al. [23]
Sensitivity of testing	71% *	Padhye [24]
	97.8% **	Taiwan CDC
Current universal testing capacity (% of total population per day)	0.05%, 0.5%, 1%	Ministry of Health and Welfare, Taiwan [25]; United States CDC [26]; United Kingdom Government [27]; OWID [28]
Projected universal testing capacity (% of total population per day)	5%, 10%	Cherif et al. [29]

* the ancestral SARS-Cov-2 virus, ** SARS-CoV-2 Omicron variant.

**Table 2:** Recommendation Strategies.

Situation	Recommendation
High populations with low COVID-19 prevalence	strategies II: contact tracing + symptomatic screening
Low populations with low COVID-19 prevalence	strategies II: contact tracing + symptomatic screening
High populations with high COVID-19 prevalence	strategies III: contact tracing + symptomatic screening+ universal testing
Low populations with high COVID-19 prevalence	strategies III: contact tracing + symptomatic screening+ universal testing

## Data Availability

The data supporting the findings can be found in the main paper.

## Supplementary Materials

The following supporting information can be downloaded at: https://www.mdpi.com/article/10.3390/tropicalmed7110376/s1, Figure S1: Incubation period, onset-to-isolation interval and serial interval distributions; Figure S2: Five possible transmission outcomes; Figure S3: Simulated total number of cases under symptomatic screening alone; Figure S4: Time to outbreak control with 70% symptomatic screening; Figure S5: Time to outbreak control with 50% symptomatic screening; Figure S6: Time to outbreak control with 30% symptomatic screening; Table S1: Daily increase of COVID-19 cases during the simulated days 1–40 in a low prevalence country (initial case number = 20); Table S2: Daily increase of COVID-19 cases under strategy III’s current and projected universal testing capacities in a low prevalence country (initial case number = 20); Table S3: Daily increase of COVID-19 cases with contact-tracing at 40% success rate in a low prevalence country (initial case number = 20); Table S4: Daily increase of COVID-19 cases with contact-tracing at 0% success rate in a low prevalence country (initial case number = 20); Table S5: Daily increase of COVID-19 cases during the simulated days 1–40 in a high prevalence country (initial case number = 200); Table S6: Daily increase of COVID-19 cases under strategy III’s current and projected universal testing capacities in a high prevalence country (initial case number = 200); Table S7: Daily increase of COVID-19 cases with contact-tracing at 40% success rate in a high prevalence country (initial case number = 200); Table S8: Daily increase of COVID-19 cases with contact-tracing at 0% success rate in a high prevalence country (initial case number = 200); Table S9: Daily increase of COVID-19 cases with contact-tracing at 0%, 40%, and 80% success rate in a low prevalence country during the period of the SARS-CoV-2 Omicron variant (initial case number = 20).

## References

[1] Kucharski A.J., Russell T.W., Diamond C., Liu Y., Edmunds J., Funk S., Eggo M.R. (2020). Early dynamics of transmission and control of COVID-19: A mathematical modelling study. Lancet Infect. Dis..

[2] Cheong K.H., Wen T., Lai J.W. (2020). Relieving cost of epidemic by parrondo’s paradox: A COVID-19 case study. Adv. Sci..

[3] Lai J.W., Cheong K.H. (2020). Superposition of COVID-19 waves, anticipating a sustained wave, and lessons for the future. BioEssays.

[4] Cheong K.H., Jones M.C. (2020). Introducing the 21st century’s new four horsemen of the coronapocalypse. BioEssays.

[5] Kretzschmar M.E., Rozhnova G., Bootsma M.C., van Boven M., van de Wijgert J.H., Bonten M.J. (2020). Impact of delays on effectiveness of contact tracing strategies for COVID-19: A modelling study. Lancet Public Health.

[6] Aleta A., Martin-Corral D., Pastore y Piontti A., Ajelli M., Litvinova M., Chinazzi M., Dean N.E., Halloran M.E., Longini I.M., Merler S. (2020). Modelling the impact of testing, contact tracing and household quarantine on second waves of COVID-19. Nat. Hum. Behav..

[7] Jian S.W., Cheng H.Y., Huang X.T., Liu D.P. (2020). Contact tracing with digital assistance in Taiwan’s COVID-19 outbreak response. Int. J. Infect. Dis..

[8] Wang C.J., Ng C.Y., Brook R.H. (2020). Response to COVID-19 in Taiwan. Big Data Analytics, New Technology, and Proactive Testing. JAMA.

[9] Libin P.K., Willem L., Verstraeten T., Torneri A., Vanderlocht J., Hens N. (2021). Assessing the feasibility and effectiveness of household-pooled universal testing to control COVID-19 epidemics. PLoS Comput. Biol..

[10] Pan J., Chen Z., He Y., Liu T., Li Q., Cheng X., Xiao J., Feng H. (2022). Why Controlling the Asymptomatic Infection Is Important: A Modelling Study with Stability and Sensitivity Analysis. Fractal Fract..

[11] Gao Z., Xu Y., Sun C., Wang X., Guo Y., Qiu S., Ma K. (2021). A systematic review of asymptomatic infections with COVID-19. J. Microbiol. Immunol. Infect..

[12] Larremore D.B., Wilder B., Lester E., Shehata S., Burke J.M., Hay J.A., Tambe M., Mina M.J., Parker R. (2021). Test sensitivity is secondary to frequency and turnaround time for COVID-19 screening. Sci. Adv..

[13] BALLOTPEDIA Arguments about Universal or Mass Testing for COVID-19 before the Economy Can Reopen. https://ballotpedia.org/Arguments_about_universal_or_mass_testing_for_COVID-19_before_the_economy_can_reopen.

[14] Hellewell J., Abbott S., Gimma A., Bosse N.I., Jarvis C.I., Russell T.W., Munday J.D., Kucharski A.D., Edmunds W.J. (2020). Feasibility of controlling COVID-19 outbreaks by isolation of cases and contacts. Lancet Glob. Health.

[15] Tsou H.H., Cheng Y.C., Yuan H.Y., Hsu Y.T., Wu H.Y., Lee F.J., Hsiung C.A., Chen W.J., Sytwu H.K., Wu S.I. (2020). The effect of preventing subclinical transmission on the containment of COVID-19: Mathematical modeling and experience in Taiwan. Contemp. Clin. Trials.

[16] Burki T.K. (2022). Omicron variant and booster COVID-19 vaccines. Lancet Respir. Med..

[17] Liu T., Hu J., Kang M., Rong Z., Lin L., Zhong H., Huang Q., Deng A., Huang Q., Deng A. (2020). Time-varying transmission dynamics of Novel Coronavirus Pneumonia in China. BioRxiv.

[18] Backer J.A., Klinkenberg D., Wallinga J. (2020). Incubation period of 2019 novel coronavirus (2019-nCoV) infections among travellers from Wuhan, China, 20–28 January 2020. Eurosurveillance.

[19] Helmsdal G., Hansen O.K., Moller L.F., Christiansen D.H., Petersen M.S., Kristiansen M.F. (2022). Omicron outbreak at a private gathering in the Faroe Islands, infecting 21 of 33 triple-vaccinated healthcare workers. Clin. Infect. Dis..

[20] UK Health Security Agency (2021). Omicron and Delta Serial Interval Distributions from UK Contact Tracing Data. https://www.gov.uk/government/publications/ukhsa-omicron-and-delta-serial-interval-distributions-from-uk-contact-tracing-data-31-december-2021.

[21] Oran D.P., Topol E.J. (2020). Prevalence of asymptomatic SARS-CoV-2 infection. Ann. Intern. Med..

[22] Garrett N., Tapley A., Andriesen J., Seocharan I., Fisher L.H., Bunts L., Espy N., Wallis C.L., Randhawa A.K., Miner M.D. (2022). High Asymptomatic Carriage with the Omicron Variant in South Africa. Clin Infect Dis..

[23] Casey-Bryars M., Griffin J., McAloon C., Byrne A., Madden J., Evoy D.C., Collins A., Hunt K., Barber A., Butler F. (2021). Presymptomatic transmission of SARS-CoV-2 infection: A secondary analysis using published data. BMJ Open.

[24] Padhye N.S. (2021). Reconstructed diagnostic sensitivity and specificity of the RT-PCR test for COVID-19. MedRxiv.

[25] Ministry of Health and Welfare, Taiwan Adequate Testing Capacity and Precisely Locate Potentially Infected Individuals. https://covid19.mohw.gov.tw/en/cp-4788-53906-206.html.

[26] United States Centers for Disease Control and Prevention Data table for COVID-19 Viral (RT-PCR) Laboratory Test 30-Day Percent Positivity by State/Territory. https://covid.cdc.gov/covid-data-tracker/#testing_positivity30day.

[27] United Kingdom Government Testing in United Kingdom. https://coronavirus.data.gov.uk/details/testing.

[28] Our World in Data (2021). Coronavirus (COVID-19) Testing. https://ourworldindata.org/coronavirus-testing.

[29] Health Affairs Universal Testing to End the Pandemic. https://www.healthaffairs.org/do/10.1377/forefront.20201102.521193/full/.

[30] Covid Act Now What Is Incidence? Covid Act Now 2020. https://blog.covidactnow.org/what-is-covid-incidence/.

[31] Ferretti L., Wymant C., Kendall M., Zhao L., Nurtay A., Abeler-Dörner L., Bonsall D., Parker M., Fraser C. (2020). Quantifying SARS-CoV-2 transmission suggests epidemic control with digital contact tracing. Science.

[32] Johnson-León M., Caplan A.L., Kenny L., Buchan I., Fesi L., Olhava P., Alugnoa D.N., Aspinall M.G., Costanza E., Desharnais B. (2021). Executive summary: It’s wrong not to test: The case for universal, frequent rapid COVID-19 testing. EClinicalMedicine.

[33] United States Centers for Disease Control and Prevention Overview of Testing for SARS-CoV-2 (COVID-19). https://www.cdc.gov/coronavirus/2019-ncov/hcp/testing-overview.html.

