# Supplementary material for: Contribution of Testing Strategies and Contact Tracing towards COVID-19 Outbreaks Control: A Mathematical Modeling Study
Source: Trop Med Infect Dis. 2022 Nov 14;7(11):376. doi: 10.3390/tropicalmed7110376 (PMC9693367; doi:10.3390/tropicalmed7110376)
Supplement: Supplementary file 1 [file tropicalmed-07-00376-s001.zip › tropicalmed-1890584-supplementary.pdf]

Supplementary Materials

Contribution of testing strategies and contact tracing towards COVID-19 outbreaks control: A mathematical modeling study

Shu-Chen Kuo [1,†], Byron Fan [2,†], Hongye Zhu [3,†], Meng-Hsuan Wu [4,†], Fang-Jing Lee [4,†], Yu-Chieh Cheng [4], Hsiao-Yu Wu [4], Ya-Ting Hsu [4], Chao A. Hsiung [4], Shiow-Ing Wu [4], Wei J. Chen [5,6], Hung-Yi Chiou [4,7,8], Huey-Kang Sytwu [1] and Hsiao-Hui Tsou [4,9,*]

[1] National Institute of Infectious Diseases and Vaccinology, National Health Research Institutes, Zhunan 35053, Taiwan
[2] Department of Applied Mathematics, Brown University, Providence, RI 02912, USA
[3] Department of Public Health, College of Public Health, National Taiwan University, Taipei 10617, Taiwan
[4] Institute of Population Health Sciences, National Health Research Institutes, Zhunan 35053, Taiwan
[5] Center for Neuropsychiatric Research, National Health Research Institutes, Zhunan 35053, Taiwan
[6] Institute of Epidemiology and Preventive Medicine, College of Public Health, National Taiwan University, Taipei 10617, Taiwan
[7] School of Public Health, College of Public Health, Taipei Medical University, Taipei 11031, Taiwan
[8] Master's Program in Applied Epidemiology, College of Public Health, Taipei Medical University, Taipei 11031, Taiwan
[9] Graduate Institute of Biostatistics, College of Public Health, China Medical University, Taichung 40402, Taiwan
* Correspondence: tsouhh@nhri.edu.tw; Tel.: +886-37-206-166 (ext. 36181)
† These authors contributed equally to this research.

Model

A stochastic transmission model was constructed to assess the feasibility of controlling the COVID-19 outbreak through contact tracing and case isolation. We assessed the effectiveness of universal testing under the same model assumptions.

Weibull

For each COVID-19 patient, we assumed that the incubation period was a Weibull random variable *X*. The probability density function of the Weibull distribution is as follows:

$$f(x)=\begin{cases}\dfrac{k}{\lambda}\left(\dfrac{x}{\lambda}\right)e^{-\left(\frac{x}{\lambda}\right)^{k}} & x\geq 0\\ 0 & x<0\end{cases}$$

where k is the shape parameter and λ is a scale parameter. The distribution is demonstrated in Figure S1a,b.

We assumed the delay from symptom onset to isolation also follows a Weibull distribution with mean and standard deviation estimated by Liu et al. [1] and Tsou et al. [2] (see Table 1 & Figure S1d).

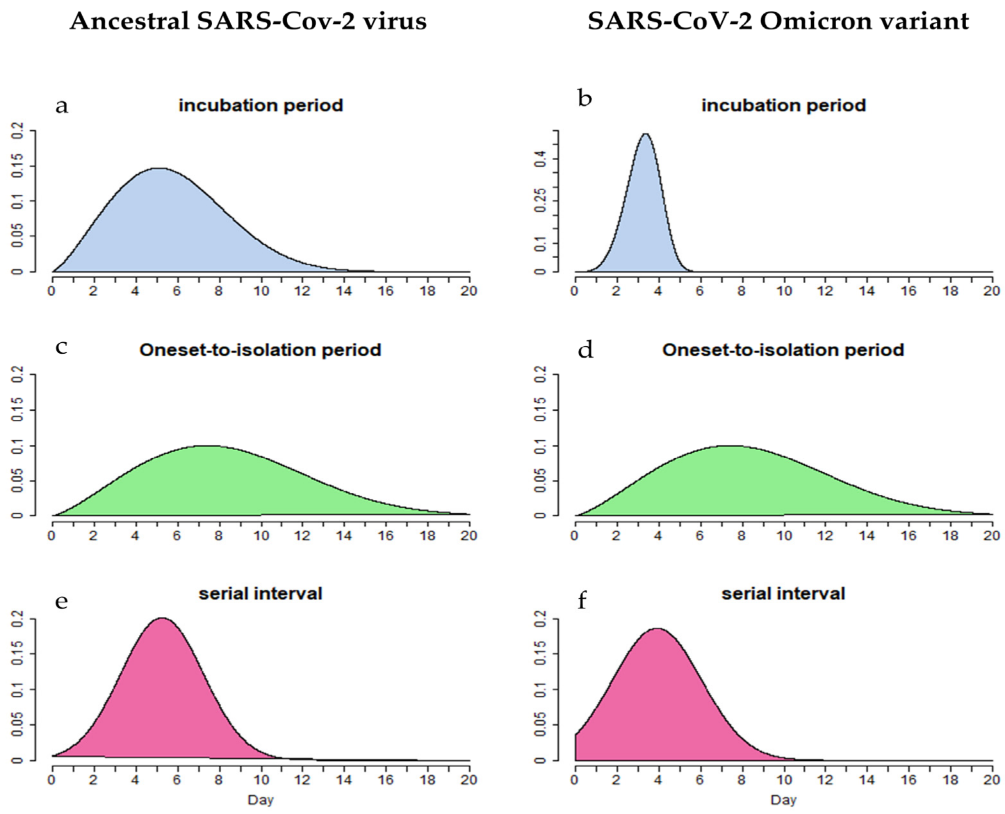

Figure S1. Incubation period, onset-to-isolation interval, and serial interval distributions during the

period of the ancestral SARS-Cov-2 virus and SARS-CoV-2 Omicron variant. (a) The incubation period for the ancestral SARS-Cov-2 virus with a mean of 5.8 days and a standard deviation of 2.6 days; (b) The incubation period for the SARS-CoV-2 Omicron variant with a mean of 3.24 days and a standard deviation of 0.8 days; (c) and (d) The delay from symptom onset to isolation was a Weibull distribution with a mean of 9.76 days and s standard deviation of 7.66 days; (e) and (f) A serial interval for each case was drawn from a skew-normal distribution with mean given by the incubation period of each case. Parameter values and references were as given in Table 1.

Negative Binomial

Note that each secondary case was created only if the infected person had not been isolated by the time of infection. We implemented a branching process model in which the number of secondary cases *Y* produced by each primary case is generated from a negative binomial distribution with a mean equal to a reproduction number *R0*. The probability density function is as follows:

$$f(y) = \frac{\Gamma(y+n)}{(\Gamma(n)y!)} p^n (1-p)^y$$

We adjusted this distribution with mean = *n(1-p)/p* equals to the reproduction number *R0*.

For each primary case, we used the negative binomial distribution to calculate the number of secondary cases produced. Then, for each secondary case, we calculated the estimated transmission date using the exposure date of the primary case, and a serial interval was generated from the skew normal distribution described below.

Serial Interval

Each new infection was assigned a serial interval *S* (time between successive cases in the transmission chain of infection) drawn from a skew-normal distribution. The corresponding probability density function is as follows:

$$f(s) = \frac{2}{\omega}\phi\left(\frac{s-\xi}{\omega}\right)\Phi\left(\alpha\left(\frac{s-\xi}{\omega}\right)\right)$$

In this function, ϕ is the standard normal probability density function, and Φ is the corresponding cumulative distribution function.

We adjusted the function with the location parameter ξ, which is drawn from the incubation period of the specific case. The scale parameter ω is assumed to be 2, and a shape parameter α is adjusted based on pre-symptomatic transmission [2]. We assumed that pre-symptomatic transmission to be 55%. The distribution is shown in Figure S1(e)

and (f).

Figure S2 demonstrated the five possible transmission outcomes from the same primary source A with different intervention procedures. Contact tracing was conducted towards cases B and F, with an 80% success rate. Symptomatic screening was applied to cases C and E. Universal testing was applied to case D. The parameter values were as given in Table 1.

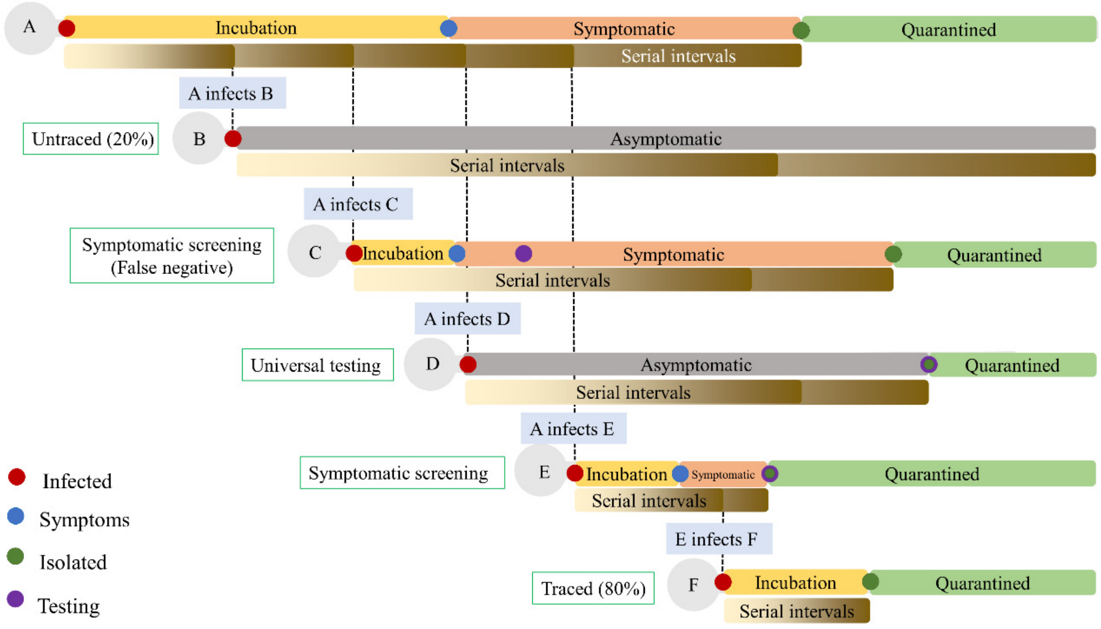

Figure S2. Five possible transmission outcomes. An example of the simulated process starting with individual A as the initial infector. Before being quarantined, individual A had the potential of infecting others during the incubation period and the delay from onset to isolation, which were both drawn using the serial interval distribution. For each of the exposed individuals, a serial interval was drawn before each case's isolation. In this example, we assume that an infected individual could be contact traced with a success rate of 80%, detected after symptomatic screening or identified by universal testing. Before isolation, each infected individual could become a secondary infector.

Subclinical cases

For each new secondary patient, we independently determined the occurrence of a subclinical case with a probability of 0.4. Note that a subclinical case would not be isolated without intervention.

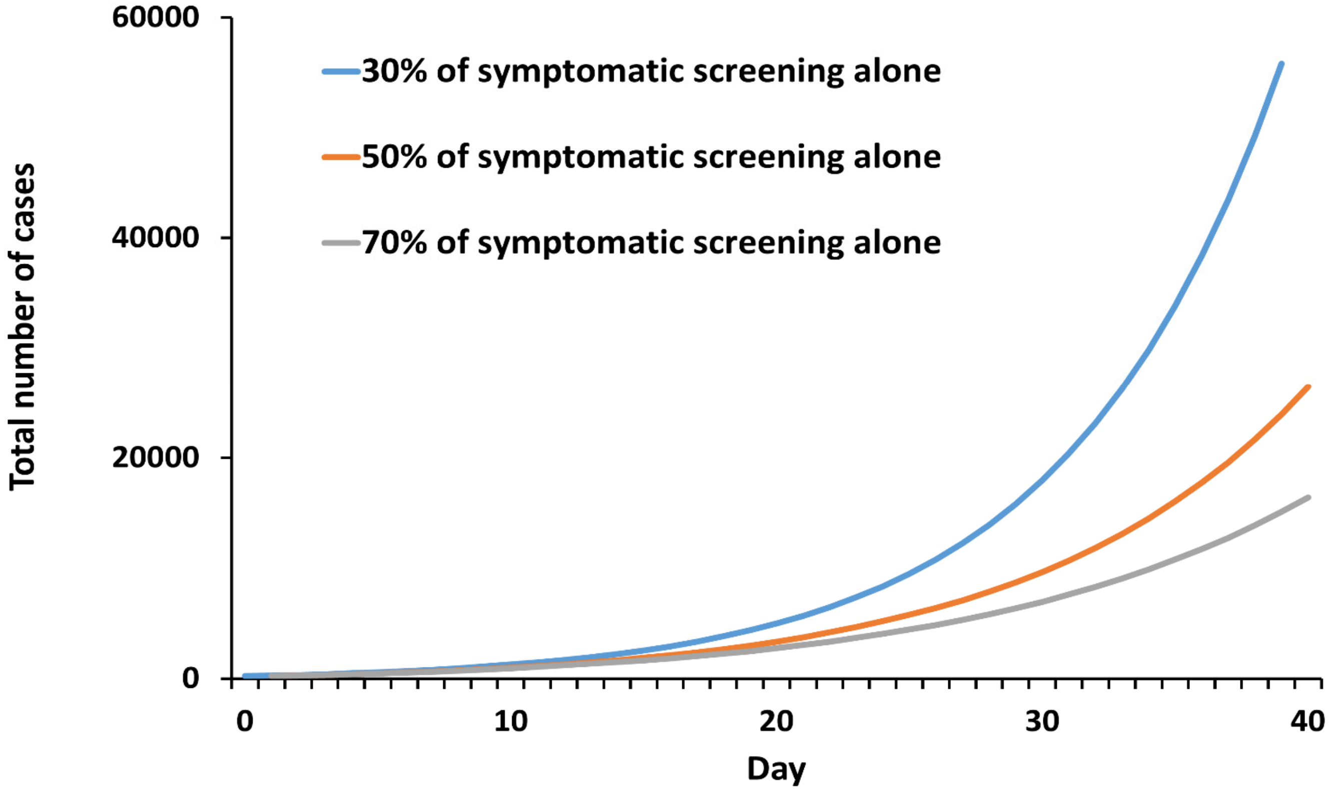

Figure S3. Simulated total number of cases under symptomatic screening alone. This figure shows that 30%, 50%, and 70% of symptomatic screening alone led to an 83.3%, 92.0%, and 94.9% case reduction, respectively, compared to the Reference in a high prevalence country.

a.

Contact tracing	Initial cases	Universal testing (%) 0	0.5	0.6	0.7	1	5	10
0%	70	–	–	–	–	–	–	–
	60	–	–	–	–	–	–	–
	50	–	–	–	–	–	–	–
	40	–	–	–	–	–	–	–
	30	–	–	–	–	–	–	–
	20	–	–	–	–	–	–	–
	10	–	–	–	–	–	–	–
40%	70	–	–	–	–	–	–	34
	60	–	–	–	–	–	–	32
	50	–	–	–	–	–	–	32
	40	–	–	–	–	–	–	30
	30	–	–	–	–	–	–	28
	20	–	–	–	–	–	37	22
	10	–	–	–	–	–	28	19
80%	70	–	–	–	40	39	26	21
	60	–	39	38	39	38	25	21
	50	–	36	39	36	37	24	19
	40	37	37	34	37	31	23	19
	30	35	35	35	31	30	21	18
	20	32	30	28	26	27	20	17
	10	23	24	23	21	22	17	15

b.

Contact tracing	Initial cases	Universal testing (%) 0	0.5	0.6	0.7	1	5	10
0%	70	–	–	–	–	–	–	–
	60	–	–	–	–	–	–	–
	50	–	–	–	–	–	–	–
	40	–	–	–	–	–	–	–
	30	–	–	–	–	–	–	–
	20	–	–	–	–	–	–	–
	10	–	–	–	–	–	–	–
40%	70	–	–	–	–	–	21	15
	60	–	–	–	–	–	19	14
	50	–	–	–	–	–	16	7
	40	–	–	–	–	–	7	7
	30	–	–	–	–	19	7	7
	20	–	7	7	7	7	7	7
	10	7	7	7	7	7	7	7
80%	70	21	19	20	19	18	15	12
	60	19	19	18	18	16	14	12
	50	7	7	7	7	7	7	7
	40	7	7	7	7	7	7	7
	30	7	7	7	7	7	7	7
	20	7	7	7	7	7	7	7
	10	7	7	7	7	7	7	7

–: The epidemic is not controlled at any time.

Figure S4. Time to outbreak control with 70% symptomatic screening. This figure shows time to outbreak control for the simulated COVID-19 pandemic with 0%, 40%, and 80% contact tracing and 70% symptomatic screening. We show the earliest day to achieve outbreak control based on (a) the strict definition and (b) the light definition.

a.

Contact tracing	Initial cases	Universal testing (%)						
		0	0.5	0.6	0.7	1	5	10
0%	70	–	–	–	–	–	–	–
	60	–	–	–	–	–	–	–
	50	–	–	–	–	–	–	–
	40	–	–	–	–	–	–	–
	30	–	–	–	–	–	–	–
	20	–	–	–	–	–	–	–
	10	–	–	–	–	–	–	–
40%	70	–	–	–	–	–	–	35
	60	–	–	–	–	–	–	35
	50	–	–	–	–	–	–	37
	40	–	–	–	–	–	–	32
	30	–	–	–	–	–	–	30
	20	–	–	–	–	–	–	25
	10	–	–	–	–	–	33	19
80%	70	–	–	–	–	–	25	22
	60	–	–	–	–	–	25	22
	50	–	–	–	–	–	26	20
	40	–	–	–	–	40	23	19
	30	–	40	40	–	34	23	18
	20	40	36	34	38	32	20	17
	10	33	26	26	25	25	16	16

b.

Contact tracing	Initial cases	Universal testing (%)						
		0	0.5	0.6	0.7	1	5	10
0%	70	–	–	–	–	–	–	–
	60	–	–	–	–	–	–	–
	50	–	–	–	–	–	–	–
	40	–	–	–	–	–	–	–
	30	–	–	–	–	–	–	–
	20	–	–	–	–	–	–	–
	10	–	–	–	–	–	–	–
40%	70	–	–	–	–	–	25	16
	60	–	–	–	–	–	22	15
	50	–	–	–	–	–	17	12
	40	–	–	–	–	–	16	7
	30	–	–	–	–	–	7	7
	20	–	–	–	–	–	7	7
	10	–	7	7	7	7	7	7
80%	70	26	24	23	22	21	15	13
	60	24	21	21	21	19	14	12
	50	22	19	19	18	17	13	7
	40	17	16	17	15	16	7	7
	30	7	7	7	7	7	7	7
	20	7	7	7	7	7	7	7
	10	7	7	7	7	7	7	7

–: The epidemic is not controlled at any time.

Figure S5. Time to outbreak control with 50% symptomatic screening. This figure shows time to outbreak control for the simulated COVID-19 pandemic with 0%, 40%, and 80% contact tracing and 50% symptomatic screening. We show the earliest day to achieve outbreak control based on (a) the strict definition and (b) the light definition.

a.

Contact tracing	Initial cases	Universal testing (%)						
		0	0.5	0.6	0.7	1	5	10
0%	70	–	–	–	–	–	–	–
	60	–	–	–	–	–	–	–
	50	–	–	–	–	–	–	–
	40	–	–	–	–	–	–	–
	30	–	–	–	–	–	–	–
	20	–	–	–	–	–	–	–
	10	–	–	–	–	–	–	–
40%	70	–	–	–	–	–	–	39
	60	–	–	–	–	–	–	38
	50	–	–	–	–	–	–	35
	40	–	–	–	–	–	–	34
	30	–	–	–	–	–	–	33
	20	–	–	–	–	–	–	29
	10	–	–	–	–	–	–	22
80%	70	–	–	–	–	–	30	23
	60	–	–	–	–	–	29	22
	50	–	–	–	–	–	28	22
	40	–	–	–	–	–	27	20
	30	–	–	–	–	–	26	21
	20	–	–	–	–	–	23	17
	10	–	–	–	40	34	18	15

b.

Contact tracing	Initial cases	Universal testing (%)						
		0	0.5	0.6	0.7	1	5	10
0%	70	–	–	–	–	–	–	–
	60	–	–	–	–	–	–	–
	50	–	–	–	–	–	–	–
	40	–	–	–	–	–	–	–
	30	–	–	–	–	–	–	–
	20	–	–	–	–	–	–	–
	10	–	–	–	–	–	–	–
40%	70	–	–	–	–	–	36	16
	60	–	–	–	–	–	33	16
	50	–	–	–	–	–	27	14
	40	–	–	–	–	–	18	7
	30	–	–	–	–	–	7	7
	20	–	–	–	–	–	7	7
	10	–	–	–	–	–	7	7
80%	70	–	39	36	37	29	16	13
	60	–	36	33	32	28	16	12
	50	–	28	27	26	22	14	7
	40	40	23	25	22	22	7	7
	30	23	18	19	15	17	7	7
	20	7	7	7	7	7	7	7
	10	7	7	7	7	7	7	7

–: The epidemic is not controlled at any time.

Figure S6. Time to outbreak control with 30% symptomatic screening. This figure shows time to outbreak control for the simulated COVID-19 pandemic with 0%, 40%, and 80% contact tracing and 30% symptomatic screening. We show the earliest day to achieve outbreak control based on (a) the strict definition and (b) the light definition.

Table S1. Daily increase of COVID-19 cases during the simulated days 1-40 in a low prevalence country (initial case number = 20).

Day	Strategy I			Strategy II			Strategy III		
	Contact tracing 0%	Contact tracing 40%	Contact tracing 80%	Contact tracing 0% + symptomatic screening	Contact tracing 40% + symptomatic screening	Contact tracing 80% + symptomatic screening	Contact tracing 0% + symptomatic screening + universal testing 0.5%	Contact tracing 40% + symptomatic screening + universal testing 0.5%	Contact tracing 80% + symptomatic screening + universal testing 0.5%
1	3.49	3.57	3.537	3.33	3.519	3.461	3.14	3.488	3.48
2	4.110	4.259	4.202	3.61	3.371	3.159	3.12	3.332	3.194
3	6.600	6.359	6.149	3.91	4.021	3.856	4.46	4.081	3.752
4	8.670	8.604	8.384	4.98	4.844	4.317	5	4.837	4.251
5	11.400	11.122	10.915	6.18	5.473	4.524	6.14	5.467	4.603
6	13.930	13.899	13.554	6.71	5.948	4.59	6.97	5.726	4.686
7	16.780	16.486	16.104	7.14	6.111	4.439	7.91	6.135	4.519
8	19.780	19.246	18.817	7.4	6.36	4.224	7.81	6.088	4.22
9	23.740	22.581	21.604	8.19	6.201	3.821	8.36	6.077	3.762
10	28.210	26.670	24.688	8.73	6.022	3.462	9.03	5.921	3.411

11	33.610	30.669	28.304	10.1	6.01	3.084	9.54	5.872	2.854
12	39.900	36.636	31.515	9.84	5.849	2.678	9.79	6.018	2.529
13	46.550	42.452	36.006	10.55	5.949	2.425	10.48	5.752	2.322
14	54.910	49.722	39.645	10.99	5.793	2.18	11.43	5.786	2.062
15	65.000	58.603	44.495	12.59	5.806	1.823	11.9	5.768	1.769
16	78.630	68.035	49.837	13.45	5.76	1.681	12.58	5.838	1.623
17	95.030	79.878	56.000	14.43	5.793	1.446	13.73	5.676	1.396
18	111.830	92.928	62.546	14.72	5.696	1.315	14.67	5.754	1.249
19	131.960	108.435	70.017	15.82	5.684	1.134	15.47	5.637	1.185
20	159.050	126.826	78.480	17.67	5.523	1.083	15.71	5.651	1.004
21	189.470	148.609	88.639	18.81	5.552	0.925	16.83	5.459	0.938
22	229.590	173.290	99.045	20.13	5.471	0.842	17.95	5.598	0.914
23	271.630	201.954	111.796	20.92	5.653	0.754	19.64	5.413	0.799
24	321.370	236.490	125.150	22.87	5.593	0.654	19.31	5.473	0.686
25	381.550	276.791	140.952	24.36	5.575	0.585	20.63	5.444	0.632
26	456.860	322.609	159.408	25.41	5.64	0.536	22.39	5.459	0.506
27	543.750	377.934	179.552	26.53	5.63	0.451	24.26	5.198	0.454
28	647.870	440.458	199.810	29.55	5.549	0.428	25.07	5.36	0.452
29	773.520	514.422	224.744	30.53	5.621	0.42	26.69	5.277	0.448
30	922.880	602.978	251.679	33.51	5.435	0.313	29.76	5.183	0.37
31	1096.110	704.574	283.064	34.37	5.501	0.292	30.86	5.207	0.343
32	1304.590	825.268	318.315	36.44	5.425	0.232	31.8	5.09	0.305
33	1551.260	961.319	356.528	39.84	5.412	0.241	33.74	5.017	0.223

34	1846.070	1125.413	400.521	40.07	5.488	0.191	36	5.008	0.252
35	2205.540	1315.543	449.597	43.57	5.405	0.184	37.13	4.896	0.232
36	2623.270	1534.797	505.062	47.3	5.271	0.156	41.33	5.02	0.158
37	3128.320	1794.885	567.681	49.92	5.284	0.15	43.96	4.967	0.167
38	3720.780	2098.101	637.334	53.41	5.301	0.12	45.26	4.88	0.152
39	4431.630	2451.633	715.001	56.92	5.016	0.123	49.27	4.891	0.1
40	5271.670	2865.289	802.591	60.98	5.192	0.109	51.46	4.951	0.094

Note: Gray areas indicates the days when the daily case number increase is lower than one.

Table S2. Daily increase of COVID-19 cases under strategy III's current and projected universal testing capacities in a low prevalence country (initial case number = 20).

Day	Current universal testing capacities			Projected universal testing capacities	
	Contact tracing 80% + symptomatic screening + universal testing 0.05%	Contact tracing 80% + symptomatic screening + universal testing 0.5%	Contact tracing 80% + symptomatic screening + universal testing 1%	Contact tracing 80% + symptomatic screening + universal testing 5%	Contact tracing 80% + symptomatic screening + universal testing 10%
1	3.495	3.558	3.506	3.41	3.557
2	3.385	3.264	3.145	3.03	3.156
3	3.816	3.739	3.769	3.593	3.553
4	4.317	4.141	4.25	3.999	3.931
5	4.759	4.386	4.605	4.196	4.24
6	4.671	4.591	4.573	4.311	4.006
7	4.692	4.499	4.336	4.159	3.826
8	4.203	4.141	4.035	3.666	3.386
9	3.741	3.855	3.675	3.201	2.928
10	3.313	3.393	3.149	2.819	2.424
11	2.905	2.971	2.8	2.461	2.035
12	2.688	2.627	2.54	2.157	1.79
13	2.423	2.39	2.352	1.829	1.476
14	2.167	2.221	1.92	1.656	1.26
15	1.838	1.898	1.685	1.437	1.044
16	1.574	1.64	1.49	1.229	0.837
17	1.395	1.449	1.311	1.074	0.675

18	1.284	1.284	1.158	0.903	0.644
19	1.214	1.16	1.076	0.798	0.524
20	1.167	1.003	0.895	0.653	0.432
21	0.958	0.979	0.868	0.607	0.389
22	0.814	0.813	0.847	0.516	0.344
23	0.741	0.733	0.687	0.419	0.282
24	0.693	0.608	0.596	0.331	0.243
25	0.615	0.579	0.525	0.303	0.216
26	0.607	0.472	0.477	0.265	0.173
27	0.501	0.401	0.425	0.225	0.129
28	0.406	0.371	0.403	0.204	0.129
29	0.38	0.34	0.335	0.168	0.101
30	0.372	0.299	0.326	0.147	0.094
31	0.283	0.283	0.303	0.132	0.077
32	0.253	0.271	0.247	0.119	0.044
33	0.216	0.208	0.229	0.108	0.049
34	0.27	0.203	0.22	0.102	0.025
35	0.214	0.141	0.203	0.095	0.027
36	0.201	0.14	0.185	0.06	0.024
37	0.17	0.139	0.16	0.066	0.021
38	0.184	0.118	0.136	0.06	0.021
39	0.158	0.105	0.108	0.034	0.016
40	0.157	0.105	0.093	0.047	0.023

Note: Gray areas indicate the days when the daily case number increase is lower than one.

Table S3. Daily increase of COVID-19 cases with contact-tracing at 40% success rate in a low prevalence country (initial case number = 20).

Day	Contact tracing 40% + symptomatic screening + universal testing 5%	Contact tracing 40% + symptomatic screening + universal testing 10%	Contact tracing 40% + symptomatic screening + universal testing 15%	Contact tracing 40% + symptomatic screening + universal testing 20%
1	3.71	3.77	3.54	3.72
2	3.16	3.38	2.94	2.82
3	3.65	3.53	2.92	2.82
4	4.27	3.58	2.66	2.65
5	4.65	3.39	3.32	3.01
6	4.18	3.29	2.82	3.19
7	4.6	3.51	2.8	2.3
8	4.46	3.03	2.47	2.32
9	4.05	2.69	2.04	1.48
10	3.6	2.5	1.76	1.51
11	3.38	2.15	1.38	1.23
12	3.96	1.93	1.33	0.97
13	3.49	1.42	1.13	0.99
14	2.97	1.53	0.93	0.65
15	3.13	1.21	0.97	0.62
16	3.13	1.05	0.67	0.5
17	3.04	0.85	0.68	0.59
18	2.65	0.88	0.64	0.3
19	2.65	0.65	0.43	0.32
20	2.54	0.51	0.28	0.2
21	2.2	0.77	0.37	0.23
22	2.04	0.51	0.3	0.12
23	2.07	0.39	0.15	0.14
24	1.91	0.4	0.18	0.13
25	1.74	0.47	0.1	0.08
26	1.79	0.37	0.17	0.12
27	1.5	0.34	0.18	0.07
28	1.84	0.28	0.13	0.04

29	1.61	0.3	0.04	0.03
30	1.3	0.21	0.09	0.05
31	1.35	0.25	0.08	0.03
32	1.11	0.13	0.12	0.03
33	1.27	0.15	0.06	0.02
34	1.18	0.21	0.08	0.03
35	1.11	0.15	0.06	0.06
36	0.92	0.1	0.03	0
37	1.15	0.09	0.04	0.02
38	0.72	0.15	0.07	0
39	0.94	0.19	0.02	0.02
40	1.12	0.08	0.04	0.01

Note: Gray areas indicate the days when the daily case number increase is lower than one.

Table S4. Daily increase of COVID-19 cases with contact-tracing at 0% success rate in a low prevalence country (initial case number = 20).

Day	Contact tracing 0% + symptomatic screening + universal testing 5%	Contact tracing 0% + symptomatic screening + universal testing 10%	Contact tracing 0% + symptomatic screening + universal testing 15%	Contact tracing 0% + symptomatic screening + universal testing 20%
1	3.67	3.34	3.41	3.59
2	3.39	3.12	2.88	2.79
3	4.22	3.61	3.37	3.16
4	4.46	3.84	3.63	3.36
5	5.65	4.27	3.38	3.19
6	5.73	4.51	4.14	3.63
7	6.2	4.09	3.36	3.17
8	6.47	4.1	3.28	3.06
9	6.63	3.88	3.02	2.95
10	6.59	4.15	2.83	3.12
11	6.14	3.74	2.54	2.19
12	6.5	3.92	2.56	1.95
13	6.57	3.88	2.34	2.45
14	6.73	3.48	2.41	2.12
15	6.56	3.54	2.4	1.88
16	7.14	3.06	1.98	1.63
17	6.78	3.13	1.86	1.48
18	7.13	2.8	1.51	1.57
19	7.35	3.26	1.72	1.26
20	6.99	3.04	1.66	1.16
21	7.21	2.92	1.62	1.31
22	7.36	2.8	1.45	1.16
23	7.3	2.55	1.33	1.04
24	7.32	2.34	1.26	0.88
25	7.79	2.69	1.32	0.84
26	8.13	2.65	1.17	0.71

27	7.56	2.67	1.02	0.8
28	7.74	2.57	0.93	0.66
29	7.71	2.58	0.88	0.59
30	7.75	2.87	0.92	0.55
31	7.96	2.46	0.8	0.4
32	7.46	1.99	0.8	0.38
33	7.97	2.27	0.69	0.46
34	8.21	2.34	0.58	0.34
35	8.41	2.31	0.7	0.35
36	8.23	2.04	0.61	0.32
37	8.16	1.91	0.44	0.36
38	8.98	1.96	0.63	0.21
39	8.71	1.81	0.48	0.28
40	9.21	1.79	0.57	0.26

Note: Gray areas indicate the days when the daily case number increase is lower than one.

Table S5. Daily increase of COVID-19 cases during the simulated days 1-40 in a high prevalence country (initial case number = 200).

Day	Strategy I			Strategy II			Strategy III		
	Contact tracing 0%	Contact tracing 40%	Contact tracing 80%	Contact tracing 0% + symptomatic screening	Contact tracing 40% + symptomatic screening	Contact tracing 80% + symptomatic screening	Contact tracing 0% + symptomatic screening + 0.5 universal testing	Contact tracing 40% + symptomatic screening + 0.5 universal testing	Contact tracing 80% + symptomatic screening + 0.5 universal testing
1	32.8	22.4	29	29	29.6	24	22.2	21.8	27.6
2	48.2	40.8	46.6	34	33.6	27.2	31.6	34.4	34.8
3	61.2	64.4	62.6	40.4	44.2	37.4	40.4	41.6	37.6
4	94.2	86.6	89.2	48.2	53	40.6	47.4	44.8	42.8
5	116.6	108.8	108.4	54.6	54.2	42.4	59	53.4	48.2
6	147	132.2	131.4	67.8	64.2	45.2	65	50.6	48.8
7	176.2	162.4	151.6	73.2	61.2	46.6	76.6	54.4	47.4
8	209.6	195.6	177.8	65.8	64.4	40.2	74.4	60.6	42.6
9	259.8	222.6	225.6	84.2	66.4	37.2	77	55.6	38.2
10	307.2	267.4	244.4	85.8	69	30.4	81.2	54.8	30.2
11	364.6	307.4	278.2	93.8	65.8	36	78.6	47.4	31
12	428.4	354.2	321.8	101	64.6	27.6	79.8	47.8	23.6
13	532.6	430.2	346	96.2	69.4	20.2	91.6	56.8	25.4
14	646.4	500	401.2	108.4	70.8	18	95.6	59.8	22.4
15	756.6	580.8	446.4	110.6	77.2	17.2	97.6	51.4	18.4
16	886.8	683.6	486.6	130.4	68.8	15.6	101.4	52.6	14.2

17	1069	812.8	574	131.6	74	13	106.6	43.2	12
18	1294.2	942	624.4	138.6	67.4	13	116.8	39.2	12
19	1563	1117.8	712.4	155.2	62	9.8	130.6	44	10.4
20	1801.6	1290.8	808.2	168.4	59.4	6.2	131.2	45.2	8
21	2102.4	1509	876.8	161	64.4	9.2	142	48.8	4.8
22	2626	1779.4	1003	179	68.6	10.2	134.4	53.2	7.8
23	3015.4	2083	1107.2	189.8	72.8	7.8	151	41.4	6
24	3675.2	2372.8	1236	206.6	54.4	6.8	165.4	48.6	5.8
25	4314.4	2844.6	1396.4	226.8	71.8	6.6	168	34.8	3.2
26	5159.4	3322	1553.2	223.6	64.8	7.6	187	42.4	5.6
27	6109.6	3793.8	1767.4	265	68.2	5.2	186.2	38.4	6
28	7295	4524	1967.6	278.2	63.6	6	198.4	39.8	4.6
29	8709.6	5321.6	2212.8	294.2	70.6	3	219.8	37	3
30	10360.6	6169.8	2445.6	300.6	71.6	2.8	218.6	31.6	2.6
31	12302.2	7222.4	2757	334	67.2	2.6	233.6	40.2	2.6
32	14759.6	8365	3039	352.2	72.4	2.4	247.4	35.6	2.4
33	17619.6	9865.2	3444	366.6	71.8	2.2	257.8	42.8	1.8
34	20983.6	11613.8	3874.2	402	64.8	2.4	277.6	44.2	1.8
35	25065	13466.2	4366	422	62.2	3.8	296.6	38.8	1.6
36	29720.2	15823	4920.2	443.6	60.2	3.4	312.2	46.2	1.6
37	35425.4	18510	5469.8	480.2	60.4	2.2	323	46	1
38	42200.8	21574.4	6199	506.4	67.2	0.8	329	34.4	0.8
39	50072	25411	6976.8	550.4	66.2	0.6	338.8	35	0.4

40	60036.4	29634.4	7904.2	559.8	62.4	1.8	366.6	37.8	0.4

Note: Gray areas indicate the days when the daily case number increase is lower than one.

Table S6. Daily increase of COVID-19 cases under strategy III's current and projected universal testing capacities in a high prevalence country (initial case number = 200).

Day	Current Universal Testing Capacity			Projected Universal Testing Capacity	
	Contact tracing 80% + symptomatic screening + universal testing 0.05%	Contact tracing 80% + symptomatic screening + universal testing 0.5%	Contact tracing 80% + symptomatic screening + universal testing 1%	Contact tracing 80% + symptomatic screening + universal testing 5%	Contact tracing 80% + symptomatic screening + universal testing 10%
1	26.5	25.7	25.1	22.3	27
2	34.3	31.6	31.5	28.2	29.3
3	41.2	39.1	45.1	36.1	30
4	47.9	42.8	43.5	37.4	33.5
5	50	48.6	47.3	38	30.4
6	58.6	49.1	51.1	45.1	30.6
7	55.9	47.9	51.4	38.2	28
8	57.1	50.3	49.2	32.7	22.9

9	57.6	50.3	49.4	28.1	15
10	56	49.8	43.5	23.2	13.4
11	55.4	43.8	44.4	20.1	12
12	57.9	46.9	38.6	15.6	5.8
13	59.1	45.9	40.8	13.4	4.9
14	62	44.7	37.8	14.5	3.6
15	55.3	49	32.6	10	2.5
16	57.7	38.2	35	8.1	2.3
17	62.1	44.5	36	9	1.7
18	60.5	41.8	32	8.9	1.8
19	56.4	39.1	30.7	4.9	0.9
20	55.9	45	29.3	5.8	1.2
21	57.6	42.9	29.5	3.3	0.8
22	55.6	38.4	28.4	3.5	0.9
23	52.4	39.9	26.1	3.8	0.8
24	49.1	34.7	26.5	2.3	0.2
25	53.7	38.6	21.9	1.8	0.3
26	56.7	34.7	23.3	1.9	0.2
27	56.1	36.3	22.2	1.8	0.3
28	53.2	36.3	21.5	1.6	0.5
29	51.5	38.4	21.9	0.6	0
30	50.5	35.9	21.3	0.8	0
31	50.9	29.4	20.1	0.3	0

32	53.3	35.2	17.9	0.5	0
33	50.2	32.5	19.3	0.3	0
34	51.8	30.6	15.9	0.2	0
35	51.1	32	16	0.3	0.1
36	51.1	30.5	15.6	0.3	0.1
37	46.3	30.3	15.5	0.1	0
38	53.2	30.2	18.5	0.1	0
39	48.8	26.5	14.7	0.1	0
40	53.1	32.6	14.1	0.3	0

Note: Gray areas indicate the days when the daily case number increase is lower than one.

Table S7. Daily increase of COVID-19 cases with contact-tracing at 40% success rate in a high prevalence country (initial case number = 200).

Day	Contact tracing 40%+ symptomatic screening + universal testing 5%	Contact tracing 40%+ symptomatic screening + universal testing 10%	Contact tracing 40%+ symptomatic screening + universal testing 15%	Contact tracing 40%+ symptomatic screening + universal testing 20%
1	25.1	24.4	24.6	23.2
2	30.4	29	26.1	27.7
3	35.8	31.7	31.1	29.4
4	38.3	31.7	30.8	25.9
5	41.1	35.9	32.4	29.5
6	45.4	34.4	30.5	25.6
7	41.9	33.3	27.9	23.4
8	39.6	31.5	26.6	20.6
9	39.3	28.4	21.6	19.1
10	40	26	18.9	12.2
11	41.6	22.6	16.6	12
12	39.4	22.8	12	8.5
13	39.4	22.8	10	9.7
14	40.8	19.6	11.8	6.3
15	33.2	16.7	10.1	6.5
16	35.2	16.1	8.4	5.4
17	30.2	16.1	7	4.4
18	35	12.6	5.8	3.6
19	32.1	12	3.6	2.7
20	32.1	10.6	4.8	1.7
21	33.8	10.2	3.6	1.4
22	26.6	11.6	3.6	2
23	28.6	9.6	1.8	2.3
24	28	8.6	2	1.5
25	24.1	9.4	3.2	0.7
26	23.4	6	2.3	0.5
27	24.5	7	1.4	1
28	23.2	6.6	1.5	0.3
29	19.2	6.9	0.9	0.6
30	21.5	4.7	0.8	0.4
31	20.8	4.2	0.6	0.2

32	22.1	4.5	1	0.3
33	22.4	3.9	0.4	1
34	20.1	4.2	0.5	0.5
35	19.1	4.5	0	0.8
36	17.1	5	0.2	0.4
37	16.7	3.9	0.2	0.3
38	17.8	4	0.2	0.5
39	17.6	2.4	0.5	0.5
40	17.3	2.5	0.3	0.1

Note: Gray areas indicate the days when the daily case number increase is lower than one.

Table S8. Daily increase of COVID-19 cases with contact-tracing at 0% success rate in a high prevalence country (initial case number = 200).

Day	Contact tracing 0% + symptomatic screening + universal testing 5%	Contact tracing 0% + symptomatic screening + universal testing 10%	Contact tracing 0% + symptomatic screening + universal testing 15%	Contact tracing 0% + symptomatic screening + universal testing 20%
1	26.5	25.1	26.9	26.4
2	33.2	31.4	27.8	30.8
3	38.3	36.8	32.7	31
4	45.5	43.9	35.3	31.4
5	54.2	43.6	39.5	32.3
6	55.7	42.4	38.4	34.4
7	61.3	40.3	37.7	30.3
8	61.6	40	42.5	32.2
9	57.3	42.6	37	29.5
10	61.6	40.3	34.2	26.7
11	61.9	41.4	31.5	23.1
12	62.3	40.2	28.8	22.8
13	61.3	39.3	27	24.1
14	64.9	35.8	28.2	21.6
15	63.5	38.3	26.4	18.2
16	59.7	34	23.9	17
17	64.4	36.8	20.6	15
18	58.9	32	20.2	16
19	64.1	35.3	21	12.8
20	59.5	32.7	20	15.6

21	63.4	30.8	20.1	13
22	64.5	26.9	17.9	11.1
23	59.8	29.1	16.4	10
24	59.7	26.6	14	9.9
25	64.4	27.2	15.7	9.3
26	66.5	26.6	14.7	8.8
27	71.4	28.1	12.2	8.2
28	64.7	22.7	11.4	7.1
29	68.5	24.7	11.1	7.2
30	70	21.1	12.1	6.7
31	72.9	22.3	10.9	5.4
32	69.5	21.3	13.1	4.4
33	75.5	20.3	10.3	4.4
34	72.3	19.6	11.2	4.9
35	74.9	18	9.6	3.2
36	73.1	15.6	9.9	3.4
37	71.4	20.4	8.9	3.7
38	72.3	17.5	9	3
39	81.3	14.1	9.2	2
40	80.7	13.8	8.2	3.4

Table S9. Daily increase of COVID-19 cases with contact-tracing at 0%, 40%, and 80% success rate in a low prevalence country during the period of the SARS-CoV-2 Omicron variant (initial case number = 20).

Day	Contact tracing 0% + symptomatic screening + universal testing 0.5 %	Contact tracing 40% + symptomatic screening + universal testing 0.5%	Contact tracing 80% + symptomatic screening + universal testing 0.5%
1	65.6	68.3	55.1
2	67.2	53.7	22.6
3	75.1	42.7	14.7
4	91	25.2	11.8
5	107.8	18.2	5.7
6	113.2	10.7	1.3
7	111.8	10.3	2.5
8	117.2	6.8	1

9	109.9	4.8	0.4
10	121.7	5	0.2
11	124.1	3.1	0
12	121.5	1.5	0
13	135	0.8	0
14	133.9	0.4	0
15	140.2	0.9	0
16	144.7	0.1	0
17	152	0	0
18	150.2	0.1	0
19	156.3	0	0
20	174	0	0
21	185.3	0	0
22	194.9	0	0
23	205.2	0	0
24	218	0	0
25	235.8	0	0
26	251.1	0	0
27	264.5	0	0
28	287.2	0	0
29	311.7	0	0
30	316	0	0
31	333.4	0	0
32	346.7	0	0
33	352.9	0	0
34	351.8	0	0
35	374.8	0	0
36	387.3	0	0
37	410.6	0	0
38	447.5	0	0
39	448.6	0	0
40	490.2	0	0

Note: Gray areas indicate the days when the daily case number increase is lower than one.

References

1. Liu, T.; Hu, J.; Kang, M.; Rong, Z.; Lin, L.; Zhong, H.; Huang, Q.; Deng, A.; Huang, Q.; Deng, A.; et al. Time-varying transmission dynamics of Novel Coronavirus Pneumonia in China. *BioRxiv* **2020**. https://doi.org/10.1101/2020.01.25.919787.
2. Tsou, H.H.; Cheng, Y.C.; Yuan, H.Y.; Hsu, Y.T.; Wu, H.Y.; Lee, F.J.; Hsiung, C.A.; Chen, W.J.; Sytwu, H.K.; Wu, S.I.; et al. The effect of preventing subclinical transmission on the containment of COVID-19: Mathematical modeling and experience in Taiwan. *Contemp. Clin. Trials* **2020**, *96*, 106101.
